# Aerobic Exercise Decreases Negative Affect by Modulating Orbitofrontal-Amygdala Connectivity in Adolescents

**DOI:** 10.3390/life11060577

**Published:** 2021-06-18

**Authors:** Li-Kun Ge, Zhuoer Hu, Weiwen Wang, Parco M. Siu, Gao-Xia Wei

**Affiliations:** 1 CAS Key Laboratory of Mental Health, Institute of Psychology, Beijing 100101, China; gelk@psych.ac.cn (L.-K.G.); huzhuoer19@mails.ucas.ac.cn (Z.H.); wangww@psych.ac.cn (W.W.); 2 Department of Psychology, University of Chinese Academy of Sciences, Beijing 100101, China; 3 Sino-Danish College, University of Chinese Academy of Sciences, Beijing 100049, China; 4 Sino-Danish Center for Education and Research, Beijing 100049, China; 5 Division of Kinesiology, School of Public Health, The University of Hong Kong, Hong Kong 999077, China; pmsiu@hku.hk; 6 CAS Key Laboratory of Behavioral Science, Institute of Psychology, Beijing 100101, China

**Keywords:** acute exercise, negative affect, amygdala, orbitofrontal cortex, depression

## Abstract

Long-term negative affect in adolescence is associated with impairment in quality of life, interpersonal function, and social adaptation. Although physical exercise could decrease negative emotion, the underlying mechanism remains largely unknown. Acute exercise with controlled intensity might be a good experimental paradigm to unravel the potential neural mechanisms underlying the effects of physical exercise on negative affect. In this study, twenty-three males in late adolescence were randomly assigned to acute exercise group (AG) or control group. The experiment contained pre-test and post-test session interleaved with 30-min moderate-intensity exercise or seated rest. In each session, a resting-state fMRI scanning was conducted followed by completing Positive and Negative Affect Schedule and Profile of Mood State. Bilateral amygdala was used as seed region to calculate t voxel-wised functional connectivity (FC) of amygdala to whole brain. The results demonstrated, for the first time, that AG exhibited increased FC between right amygdala and right orbital frontal cortex. Significantly decreased negative affect was also observed in AG. Moreover, the increased rOFC-amygdala FC was also associated with the decreased depression score. Our findings suggest that exercise-induced decreased negative affect might be modulated by functional interactions of amygdala with both cognitive control and limbic networks, which offers a meaningful insight for clinical treatment and prevention of emotional disorders in late adolescence.

## 1. Introduction

Adolescence is a unique time with significant maturation of the brain marked by structural alterations in many limbic and cortical regions. During this period, individuals are prone to suffer from emotional disorders [1,2], such as generalized anxiety disorder and major depressive disorder due to academic stress and cognitive bias towards negative life events, which lead to impaired cognitive function, poor social adaptation and even self-injury and suicide [3]. According to the 2019–2020 report on national mental health development in China, in 2020, the detection rate of adolescent depression was 24.6%, which increased with the school grade. Notably, the average level of anxiety and depression among Chinese people between 18 and 24 is higher than at any subsequent age in adulthood [4]. Children in late adolescence, between the ages of 15 and 19, experience the stress of maturation, academic achievement expectations and changes in social roles, during which negative affect could be easily evoked [5]. Therefore, it is of great significance to develop an evidence-based regimen for the prevention and treatment of emotional disorders in adolescents.

Previous studies have shown that acute exercise can yield a reduction in anxiety, tension, depression, anger, and confusion among adolescents and adults [6,7]. Though all exercise appears to be effective, physical exercise at moderate intensity may offer a more pronounced reduction of negative emotions. Specifically, 30-min of moderate-intensity acute aerobic exercise could significantly decrease the state of anxiety [8]. Another study found that acute aerobic exercise with moderate intensity was more effective in improving negative emotions induced by unpleasant scene pictures, compared with low-intensity acute aerobic exercise [9]. Although negative emotions could be decreased by acute exercise with moderate intensity, the neural mechanism underlying it remains largely unknown.

The amygdala, a diamond-shaped brain region structurally associated with the striatum, frontal lobe, and temporal lobe [10], plays a predominant role in emotional processing response to negative emotional stimuli [11]. An increasing number of clinical studies found that the patients with emotional disorders showed impaired gray matter volume in amygdala [12] and aberrant functional activities in amygdala. For instance, atrophied amygdala and decreased functional connectivity of amygdala to the cortico-striatal-pallidal-thalamic circuit were significantly observed in the patients with depression comorbid anxiety [13]. Similar findings on amygdala were also observed in the patients with major depressive disorder [14] and generalized anxiety disorder [15]. Intriguingly, some imaging studies on antidepressant treatment also found that after receiving treatment, those patients with major depressive disorder exhibited increased functional connectivity of the amygdala to the frontal cortex [16]. In addition, the development of negative affect processing in adolescents is characterized by a general reduction in amygdala reactivity and a ventral-to-dorsal shift in the medial prefrontal response to negative emotional stimuli [17]. Thus, these findings support the role of the amygdala, which might constitute a neural mechanism underlying the established effect of acute aerobic exercise on negative emotions.

Currently, only a few human studies have examined the roles of brain regions involved in negative emotions induced by acute exercise. A recent electroencephalogram (EEG) study found that 24 min of moderate-intensity exercise and high-intensity interval exercise improved participants’ mood, and this improvement was accompanied by a stronger effect of the dorsolateral prefrontal cortex on the temporal region [18]. Furthermore, a recent functional magnetic resonance imaging (fMRI) study examined the neural mechanism underlying the effect of a 15-min moderate to high-intensity treadmill running on the perception of emotional faces, which found that compared with walking, running produced significant anxiolytic effects, indicating the underlying neural mechanisms might be related to amygdala reactivity and its connectivity with the orbitofrontal cortex and insula [19]. Similarly, a functional near-infrared spectroscopy study also observed the crucial role of the amygdala and prefrontal cortex in regulating negative emotional responses during acute exercise [20]. These studies consistently observed increased functional activity of prefrontal cortex.

Therefore, it is hypothesized that acute exercise with moderate intensity could increase the functional connectivity between amygdala and other brain regions including prefrontal cortex, which was associated with the decreased negative affect. In the present fMRI study, we used resting-state fMRI (rs-fMRI) to investigate acute exercise-induced regional changes of amygdala-based voxel-wised functional connectivity in the whole brain to unravel the neural mechanism that underlies exercise-induced change of negative emotions in late adolescence.

## 2. Materials and Methods

### 2.1. Participants

A total of twenty-four healthy male adolescents aged 18–19 years who were college students were eligible to participate in the present study. All participates were recruited from a local university and were randomly assigned to either an acute exercise group (AG) or a control group (CG). All participants were screened by a health screening questionnaire to confirm the condition to safely engage in aerobic exercise, or they underwent a cardiovascular fitness test. Additional inclusion criteria were being: (1) right-handed, (2) free of neurological and psychiatric disorders, (3) not taking any medication and no history of substance abuse, (4) cognitively intact. One participant who showed macroscopic incomplete anatomical imaging was excluded from the experimental group. Further information regarding participants is summarized in Table 1.

The Institute Review Board of Institute of Psychology, Chinese Academy of Sciences approved this study. This study was performed in accordance with the ethical standards laid down in the 1964 Declaration of Helsinki and its later amendments. The procedure of the study was fully explained to the participants and informed written consent was obtained from each of them before the study.

### 2.2. Behavioral Measures

Positive and Negative Affect Schedule (PANAS) measures both positive affect (PA) and negative affect (NA), as a reliable, valid, and efficient means. The internal consistency reliabilities (Cronbach’s coefficient α) of PANAS are acceptably high, with PA of 0.86~0.90 and NA of 0.84~0.87. It consists of twenty items, 10 adjectives describing positive and 10 describing negative affect. Each item consists of a word describing an acute emotional state and is answered on a Likert scale from 1 to 5 points, with 1 being very mild and 5 being very strong [21].

The abbreviated profile of mood state (A-POMS) was developed in POMS-SF when administered in a sport setting, consisted of 40 adjectives that measured seven dimensions of emotion (tension, anger, fatigue, depression, vigor, confusion, and esteem-related affect). The reliability coefficients (Cronbach’s α) for the subscales of A-POMS ranged from 0.664 to 0.954 with a mean of 0.789. All items are rated 5-point Likert-type scale anchored with “Not at all” to “Extremely” [22].

### 2.3. Heart Rate Monitoring

Each participant’s heart rate (HR) was recorded by a Polar heart rate monitor (Sport Tester PE 3000, Polar Electro OY, Kempere, Finland) during the exercise and through the recovery state to determine the intensity of exercise and recovery level, respectively. Age-predicted maximum heart rate (APMHR = 220-age) was calculated according to the age to monitor the intensity of exercise in the intervention group. The Borg Rating of Perceived Exertion (RPE) is a simple way to quantitatively measure the intensity of exercise during physical activity. It assesses how hard the participant feels like the body is working, which is a self-report intensity measurement ranging from 6 to 20 with 6 being no effort at all and 20 being your maximum efforts. For instance, 13 is corresponding to the heart rate of 130 times per minute, indicating moderate intensity during exercise.

### 2.4. Experimental Procedure

The participants visited our laboratory individually and completed the experiment that consisted of a pre-test session (T0) and a post-test session (T1) interleaved with 30 min exercise with moderate-intensity or 30 min resting state within one day. For the pre-test sessions, all participants were informed about a brief introduction to the study and completed informed consent, demographic data as well as behavioral questionnaires. Meanwhile, a Polar HR monitor was affixed to establish resting HR during their 10 min quietly sitting in a comfortable chair from each participant. Lastly, all participants underwent the MRI scanning for about 30 min. The participants completed the treatment conditions in a counterbalanced order. During this period, AG participated in the aerobic exercise while CG completed a reading task.

### 2.5. Exercise Intervention

The acute exercise intervention was completed on a MONARK 834 cycle ergometer (Monark, Varberg, Sweden). The participants in AG engaged in aerobic exercise on a cycle ergometer for 30 min, which involved 5 min of warm-up, exercised for 20 min at work rate with an average 77 ± 4 rpm pedaling rate, and 5 min cool-down. The participants started at a workload similar to 60% of their APMHR (moderate exercise) in the warm-up. A computer connected to a bicycle was used to ensure that the work rate corresponding to 60–69% APMHR on cycling exercise. Heart rate (HR), power output (w), revolutions per minute (rpm), and the perceived exertion (measured using RPE scale; to adjust the intensity of exercise and HR) were collected every 2 min during cycling. In the control group, participants were instructed to read neutral scientific material for 30 min while recording their HR. And the participants were also asked “what do you feel about the material” right after the reading and then orally reported the mood with two questions involving “exciting” or “boring”. After exercise or reading, participants underwent MRI scans for a second time, which followed the same scanning as before, and then completed behavioral measurements in a quiet room.

### 2.6. fMRI Scans

Brain imaging was performed on a 3T Trio system (Siemens, Erlangen, Germany) with a 12-channel head matrix coil. Resting-state functional images were obtained by using an echo-planar imaging (EPI) sequence with the following scan parameters: repetition time (TR) = 2000 ms, echo time (TE) = 30 ms, flip angle (FA) = 90°, slice thickness = 3.0 mm, field of view (FOV) = 200∗200 mm^2^, voxel-size = 3.4 × 3.4 × 4.0 mm^3^, resulting in 243 brain volumes of 30 axial slices. During the rs-fMRI scans, all participants were instructed to keep their eyes closed, relax, and move as little as possible. High-resolution structural images with the resolution of 1.3 × 1.0 × 1.3 mm were acquired using a magnetization prepared rapid gradient echo (MPRAGE) three-dimensional T1-weighted sequence (TR = 2530 ms, TE = 3.39 ms, FA = 7°, slice thickness = 1.33 mm).

### 2.7. Imaging Processing

Preprocess of the structural and functional images was implemented with the Connectome Computation System (CCS: https://github.com/zuoxinian/CCS, accessed on 1 March 2020) [23], which integrates Freesurfer (version 5.1), FSL, and AFNI to provide a pipeline system for multimodal image analysis. And all individual images went through the same pipeline, including the following steps: (1) skull stripping and cortical surface reconstruction, (2) removing the first 5 EPI volumes (10 s) of each scan, (3) removing and interpolating temporal spikes, (4) correcting slice timing, (5) eliminating physiological noises and head motion, (6) intensity normalization based on 4D global mean, (7) band-pass temporal filtering (0.01–0.1 Hz), (8) removing the linear and quadratic trends, (9) registration between individual functional image and anatomical image using boundary-based transformation (BBR) [24], and (10) projecting individual time series to the standard cortical surface (subject fsaverage5 in Freesurfer 5.1).

A hypothesis-driven analysis in which the amygdala-based functional connectivity (FC) would be increased after acute exercise was performed. Using bilateral amygdala as seed regions, which were generated by the Freesurfer segmentation procedure, we extracted the mean time series of each amygdala for every participant and calculated the Pearson’s correlation coefficient (r) between the time series of the amygdala and the whole brain voxel. The correlation coefficient (r) represents the strength of the FC between regions. A Fisher r-to-z transformation was performed for the correlation coefficients to generate normalized functional connectivity z (r).

To evaluate the exercise effect on FC between the amygdala and the whole brain, a two-sample t-test was performed for all connectivity (*p* < 0.05, Bonferroni corrected), with the intracranial volume (ICV), the root mean square of the frame-wise displacement (measuring the head motion) [25] and the minimal cost BBR (the warp distortion amount of function-to-structure realignment based on BBR) as covariates. Specifically, we examined the intervention effect by calculating postFC –preFC between AG and CG. Statistical maps were corrected for multiple comparisons with the Gaussian random field (GRF) theory (*p* < 0.001 at voxel-level, *p* < 0.01 at cluster level, two-tailed).

### 2.8. Statistical Analysis

Behavioral data were analyzed using SPSS 23.0 (IBM Corp., Armonk, NY, USA). Generalized estimating equation models (GEE) (dependent function: Normal) were used to characterize the change in behavioral scores between the two groups over time for model-based estimation, with age and baseline heart rate as covariates. The core model with behavioral scores as the outcome included group (AG and CG), time (T0 and T1), and the interaction of time with group. The T0 and T1 data of each group were compared by the Wilcoxon signed-ranks test for continuous variables. Bonferroni-adjusted *p* values of less than 0.005 (0.05/10 = 0.005) were considered statistically significant. A two-tailed correlation analysis was performed to investigate the association between the behavioral scores of emotional affects and the association between the significant connectivity changes.

## 3. Results

### 3.1. The Effects of the Acute Exercise on Emotional States

**Figure 1:**
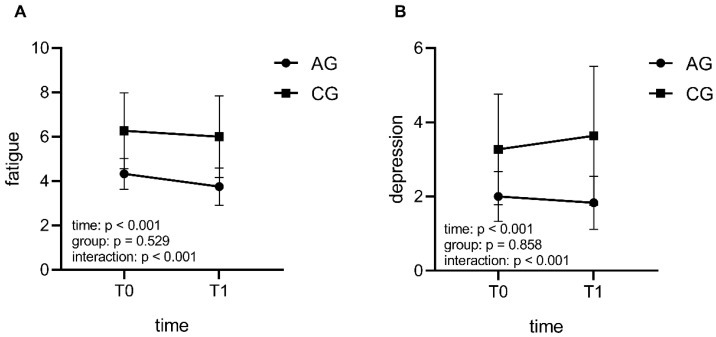
The interaction effects of intervention conditions and time (T0: pre-test session; T1: post-test session) on fatigue and depression. Compared with CG (control group), AG (acute exercise group) showed greater reductions over time in fatigue dimension (**A**) and depression dimension (**B**).

There were no significant differences both in demographics and baseline measurements (*p* > 0.05, Table 1). No participants reported that the material was exciting or boring in the CG. GEE analysis revealed the significant interaction effects in fatigue score (Wald χ^2^ = 59.339, *p* < 0.001, Figure 1A) and depression score (Wald χ^2^ = 13.098, *p* < 0.001, Figure 1B; Supplementary Material, Table S2). Specifically, AG showed a significant greater reduction in fatigue and depression scores. Regarding PANAS scores, we did not observe any significant interaction effects in both PA and NA, but AG demonstrated greater reduction in negative affect, while no significant change in CG (AG: *p* = 0.005; CG: *p* > 0.005; Supplementary Material, Table S1).

### 3.2. The Effects of Acute Exercise on Functional Connectivity

**Figure 2:**
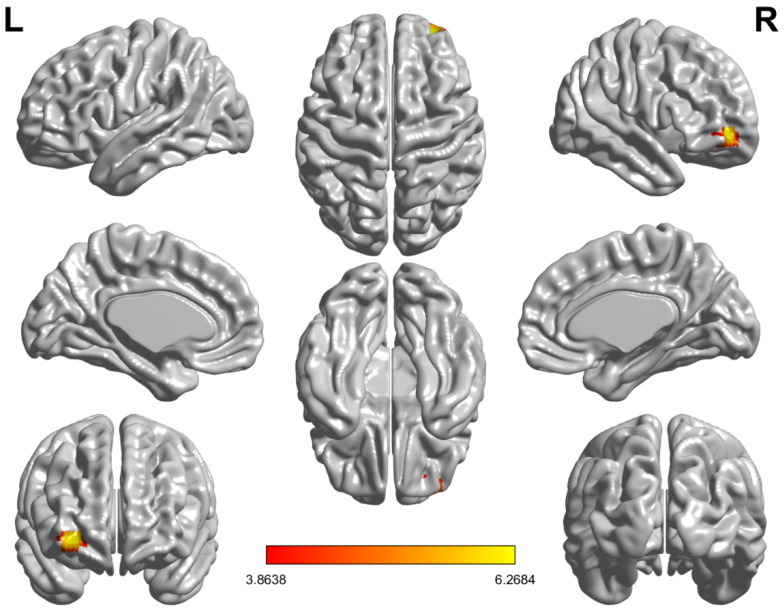
Functional connectivity between the right amygdala and the right middle frontal gyrus (orbital part)/the right superior frontal gyrus (orbital part) (right orbitofrontal cortex) was significantly increased in the acute exercise group compared to the control group.

Before the intervention, there were no significant group differences in functional connectivity between the amygdala and the whole brain between AG and CG at baseline. After the intervention, as shown in Figure 2, compared CG, AG showed significantly increased functional connectivity between the right amygdala and the right middle frontal gyrus (orbital part)/ right superior frontal gyrus (orbital part) (in one single cluster, right orbitofrontal cortex; rOFC), while there was no significant change in functional connectivity between the left amygdala and the whole brain.

Besides, we did not detect other significant changes in the functional connectivity between the amygdala and the whole brain in CG (see Supplementary Material, Table S4 for details).

### 3.3. Correlation between Functional Connectivity and Negative Emotions

**Figure 3:**
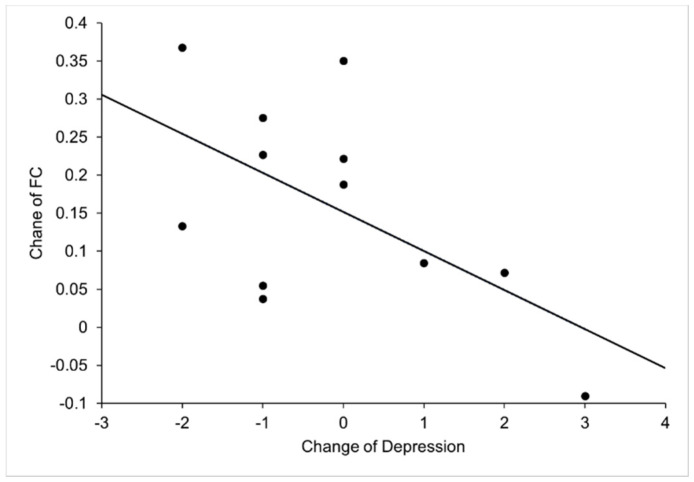
This scatterplot indicates a significant correlation between the change of depression score and the change of functional connectivity (FC) between right amygdala and right orbitofrontal cortex in AG (T1-T0; r = −0.76, *p* = 0.008, age and baseline heart rate as covariates; r = −0.776, *p* = 0.014, age, baseline heart rate and exercise heart rate as covariates).

We calculated the correlation between fMRI connectivity and scores of emotional scales with significant differences. The results demonstrated that the altered FC of the right amygdala-rOFG was significantly correlated with the changed depression score in AG (change = T1-T0; r = −0.76, *p* = 0.008, controlling for age and baseline-HR; r = −0.0776, *p* = 0.014, controlling for age, baseline-HR and exercise-HR) (Figure 3, Supplementary Material, Table S3). Additionally, we did not observe any correlation between functional connectivity and emotional scores in CG.

## 4. Discussion

The present study examined the neural mechanism underlying the effects of acute exercise with moderate intensity on negative emotions using resting functional MRI. The behavioral findings revealed that acute exercise with moderate intensity reduced the negative affect, especially fatigue and depression, of individuals in late adolescence, as indicated by PANAS and POMS. At the neuroimaging level, a significant increase was found in the FC between the right amygdala and the rOFC, including the right middle frontal gyrus (orbital part)/the right superior frontal gyrus (orbital part), after 30 min of acute exercise. The increased FC in the right amygdala with rOFC was significantly correlated with the decreased depression, suggesting that the improvement of negative emotion, especially the change of depression was associated with exercise-induced FC change in OFC-amygdala circuits.

After a single bout of acute aerobic exercise, participants reported a significant reduction in negative emotions. It is consistent with previous literature [26]. In this study, we not only observed decreased general negative emotions but also reduced depression and fatigue. Such a reduction in these specific negative emotions associated with physical exercise has been reported by a large number of studies on acute exercise [27]. One possible psychological mechanism that might contribute to distraction after acute exercise. Behavioral evidence demonstrated that distracting oneself from negative thoughts could be responsible for the mood-elevating effects of exercise [28]. Moreover, exercise could alter emotional response styles and reduce negative repetitive thinking, to promote emotional flexibility, which is the ability to recover from a negative mood [29]. And acute exercise may foster emotional flexibility and moderated the effects of the expected difficulties in emotion regulation on enduring negative emotions [29]. Last but not the least, the decreased depression and other negative affect induced by exercise is possibly associated with increased self-efficacy [30]. An acute aerobic exercise bout is sufficient to enhance exercise-related self-efficacy and produce fewer negative affective states [31]. The self-efficacy hypothesis states that the physiological effects of accomplishing a task that requires effort, such as a session of exercise, create a feeling of control that reduces negative emotions [28]. However, based on the existing literature, the exact explanations for the emotional effects of acute exercise remained largely unknown from psychological perspectives, which are warranted to be investigated in the future.

In addition to reduced negative emotions, the current study also observed significantly increased FC of amygdala to rOFC after acute aerobic exercise. It is well established that the OFC plays a crucial role in uninstructed negative emotion modulation [32]. It is responsible for conscious regulation of negative affect [33]. Further evidence suggests that OFC control the internal processing of the amygdala [34]. This subprefrontal area is co-altered to a greater extent with the activation of the amygdala response, especially when actively trying to suppress emotion through reevaluation, the greater the reappraisal task-dependent amygdala–OFC coupling, the more effective the reappraisal strategy, and the lower the level of negative affect was. [33]. Acute exercise promotes cognitive reappraisal and emotional regulation by activating the orbitofrontal cortex in young adult women [35]. Therefore, it is likely that the improvement of emotional experience induced by physical activity is related to the enhanced cognitive process caused by increased rOFC–amygdala FC.

Our results showed that the improvement of depression was significantly associated with increased FC in the right OFC-amygdala. Several studies have found that both depressed adolescents and adults showed decreased or disconnected FC between amygdala and OFC [36] compared to healthy people. The interpretation may be that OFC failed to “top-down” inhibit amygdala activity and there is an emotional processing bias with bilateral disconnections between the amygdala and the OFC, which is more severe in the right hemisphere and may be consistent with a more prominent role of the right hemisphere in emotional processing [36,37]. After antidepressant treatment, the patients’ depressive symptoms were improved significantly. Moreover, the abnormal OFC-amygdala structure and effective connectivity were normalized [37]. Therefore, it is suggested that exercise, as an effective intervention to treat depression and alternative medicine to antidepressants, is as effective as antidepressants in long-term treatment [38,39] and might lead to increased FC in brain regions implicated in major depressive disorder [40]. Here, we reasonably speculate that the effect of aerobic exercise on functional connectivity might be considered comparable to the effect of antidepressants on functional connectivity.

The aforementioned evidence has demonstrated that physical exercise could exert influences on the functions of both amygdala and OFC. The dual-mode theory postulates that affective responses to exercise are determined by the continuous interplay between cognitive parameters (from PFC to the amygdala) and interoceptive cues (from subcortical homeostatic afferents to amygdala) [20]. A greater ability to down-regulate negative emotions means a greater amygdala attenuation and greater inverse OFC/dlPFC-amygdala FC [41]. Therefore, on one hand, the OFC-amygdala FC enhancement could be observed because the exercise strengthens the activity of the OFC. Lateral OFC activity was associated with down-regulation of emotional responses, and a functional coupling of the amygdala during the negative appraisal process [42]. The prefrontal control system influences the amygdala activity in the emotional system [43] by combating the sensory input to the amygdala [20] and then affect the emotional response. On the other hand, exercise could directly decrease the activity of amygdala. Accumulative studies demonstrated the role of amygdala and the brain network involving amygdala [19,44]. A recent fMRI study used a within-subject crossover design to explore amygdala reactivity to explicit and implicit perception of emotional faces in forty young adults after 12-min aerobic exercise, which showed running elicited positive functional connectivity between amygdala and orbitofrontal cortex as well as insula [19]. Similarly, another study also found the FC between the amygdala and right anterior insula increased after high-intensity exercise intervention, while FC decreased after low-intensity exercise intervention [44]. As an important brain structure of emotion regulation, the amygdala is closely associated with negative mood states including depressive symptoms, and anxiety [45]. The right amygdala is involved in overall emotional processing, faster habitual responses, and automatic responses to emotional stimuli [45]. Moreover, the amygdala is also a gateway that links emotional regulatory sensorimotor stimuli to the central affect circuit through emotional label and regulation of the storage of emotional events in long-term memory [46]. Taking together, physical exercise could harness its role possibly by simultaneously regulating OFC and amygdala to decrease the experience of negative emotions such as depression. It also might partly contribute to the neural correlates underlying the improved negative emotion followed by a single bout of aerobic acute exercise.

Several limitations should be considered in the current study. Regarding the differences in potential hormone cycle-linked humoral factors, our participants were restricted to a specific gender (male) to exclude gender as a confounding variable in the effect of acute exercise on emotion regulation. Although this effect supported our hypothesis in this study, the sample with gender balance is needed to be examined in future research. Moreover, the results of our research may be restricted by the small sample size, as neuroimaging requires powerful statistical power; therefore, future study with larger sample size is encouraged. In addition, some previous studies have explored the relationship between acute exercise intensity and mood regulation [20], which found that high-intensity physical activity resulted in significantly more negative affect than either light or moderate-intensity physical activity [47]. Although we have confirmed in this study that moderate-intensity acute exercise can reduce negative affect, we could not generalize this effect and neural mechanism to all exercise intensity, which needs to be assessed in further investigation. Finally, given the separate function of amygdala subregions [48], our study also investigated the functional connectivity between the amygdala subregions and the whole brain, which did not observe any significant results. Our future studies will further explore the distinctive role of amygdala subregions in an exercise-induced emotional state in a larger sample size.

## 5. Conclusions

In conclusion, the findings of the present research indicated that moderate-intensity acute exercise reduced negative emotions, especially depression, which was associated with enhanced OFC-amygdala FC. From the perspective of cognitive neuroscience, the neurologic evidence of the current study supports the beneficial effects of moderate acute exercise on the regulation of negative emotions. The optimized functional connectivity between the amygdala and other cortical regions to manage negative affect induced by exercise intervention is largely attributed to this underlying neural modulation, offering a promising direction for the prevention and clinical treatment of emotional disorders, especially depression disorders, in individuals in late adolescence.

## Figures and Tables

**Table 1:** Participant’s demographics and scores of positive and negative affect at baseline.

Variables	AG (n = 12)	CG (n = 11)	*p*-Value
Age (y)	18.18 ± 0.39	18.45 ± 0.52	0.74
BMI (kg/m^2^)	21.6 ± 3.41	21.3 ± 2.65	0.79
Baseline-HR (rpm)	66.81 ± 9.52	69.70 ± 9.10	0.49
Exercise-HR (rpm)	122.58 ± 2.96	--	
Tension	4.00 ± 2.17	6.18 ± 4.71	0.18
Anger	2.00 ± 1.48	3.73 ± 4.47	0.25
Fatigue	4.33 ± 2.42	6.27 ± 5.69	0.34
Depression	2.00 ± 2.34	3.27 ± 4.94	0.43
Vigor	15.92 ± 2.81	15.36 ± 4.82	0.74
Confusion	4.58 ± 2.68	6.18 ± 5.10	0.35
Esteem	11.42 ± 2.84	11.36 ± 3.04	0.97
TMD	89.58 ± 6.36	98.91 ± 25.17	0.26
PA	32.83 ± 4.69	33.91 ± 5.49	0.62
NA	18.00 ± 3.52	19.91 ± 5.97	0.36

BMI, body mass index; HR, heart rate; TMD, total mood disturbance; PA, positive affect; NA, negative affect.

## Data Availability

Our data is not able to be made openly available because of ethics and privacy.

## Supplementary Materials

The following are available online at https://www.mdpi.com/article/10.3390/life11060577/s1, Table S1: The behavioral measures at baseline and after intervention condition in acute exercise group and control groups, Table S2: Generalized estimating equation analysis for behavioral measures, Table S3: Correlations between changes in functional connectivity and behavioral changes, Table S4: Voxel-wised functional connectivity of bilateral amygdala to other brain regions, Table S5: FC intensity and significant level before and after intervention in all participants and the acute exercise group, Table S6: Correlations between changed FC, affect scores, and HR.
