# Supplementary material for: Aerobic Exercise Decreases Negative Affect by Modulating Orbitofrontal-Amygdala Connectivity in Adolescents
Source: Life (Basel). 2021 Jun 18;11(6):577. doi: 10.3390/life11060577 (PMC8234212; doi:10.3390/life11060577)
Supplement: Supplementary file 1 [file life-11-00577-s001.zip › life-1233677-Supplementary Material.pdf]

life

MDPI

Supplementary material of Aerobic Exercise Decreases Negative Affect by Modulating Orbitofrontal-Amygdala Connectivity in Adolescents

Table S1. The behavioral measures at baseline and after intervention condition in acute exercise group and control groups.

	AG			CG		
	PRE	POST	*p*	PRE	POST	*p*
A-POMS						
Tension	4.00±2.17	3.67±2.53	0.438	6.18±4.71	4.73±4.52	0.064
Anger	2.00±1.48	1.33±1.67	0.159	3.73±4.47	3.09±4.57	0.107
Fatigue	4.33±2.42	3.75±2.93	0.254	6.27±5.69	6.00±6.10	0.180
Depression	2.00±2.34	1.83±2.48	0.672	3.27±4.94	3.64±6.20	0.739
Vigor	15.92±2.81	16.92±3.00	0.202	15.36±4.82	16.27±4.24	0.120
Confusion	4.58±2.68	3.92±2.78	0.321	6.18±5.10	5.73±4.94	0.472
Esteem	11.42±2.84	11.58±3.55	0.569	11.36±3.04	11.73±1.90	0.569
TMD	89.58±6.36	86.00±10.49	0.169	98.91±25.17	95.18±27.19	0.065
PANAS						
PA	32.83±4.69	38.17±20.23	0.663	33.91±5.49	33.27±6.23	0.473
NA	18.00±3.52	13.58±3.45	0.005	19.91±5.97	17.09±6.64	0.043

AG, acute exercise group; CG, control group; A-POMS, the abbreviated profile of mood state; TMD, total mood disturbance; PANAS, Positive and Negative Affect Schedule; PA, positive affect; NA, negative affect. Bonferroni-adjusted $p < 0.005$.

Table S2. Generalized estimating equation analysis for behavioral measures.

	Time		Group		Time*Group	
	Wald χ^2	*p*	Wald χ^2	*p*	Wald χ^2	*p*
Tension	5.363	0.021	0.714	0.398	2.031	0.154
Anger	47.568	<0.001	0.602	0.438	0.393	0.531
Fatigue	1707.105	<0.001	0.396	0.529	59.339	<0.001
Depression	209.570	<0.001	0.032	0.858	13.098	<0.001
Vigor	7.051	0.008	0.028	0.866	0.020	0.889
Confusion	9.504	0.002	0.917	0.338	0.079	0.779
Esteem	0.193	0.661	0.121	0.728	0.002	0.968
TMD	4188.235	<0.001	1.342	0.247	1.624	0.203
PA	0.557	0.455	0.067	0.795	1.020	0.313
NA	28.270	<0.001	1.218	0.270	0.478	0.489

TMD, total mood disturbance; PA, positive affect; NA, negative affect.
Bonferroni-adjusted $p < 0.005$.

Table S3. Correlations between changes in functional connectivity and behavioral changes.

		Tension	Anger	Fatigue	Depression	Vigor	Confusion	Esteem	TMD	PA	NA
AG_FC	r	-0.345	-0.147	-0.016	-0.778	0.270	-0.413	0.000	-0.449	0.067	-0.443
	p	0.329	0.686	0.966	0.008	0.450	0.236	1.000	0.193	0.855	0.200
CG_FC	r	0.247	-0.462	0.339	0.464	0.029	0.706	-0.119	0.346	-0.426	0.279
	p	0.555	0.249	0.412	0.247	0.946	0.050	0.780	0.401	0.293	0.504

AG_FC/CG_FC, the change of functional connectivity between amygdala and right orbitofrontal cortex in acute exercise group or control group;.

Table S4. Voxel-wised functional connectivity of bilateral amygdala to other brain regions.

Seed ROI	Cluster	Regions	Peak Coordinate (X Y Z)	Cluster Size (Voxels)	Peak Intensity (t value)
Right Amygdala	Cluster 1	right middle frontal gyrus (orbital part) right superior frontal gyrus (orbital part)	30 54 -6	42	6.26843
	Cluster 2	right superior frontal gyrus (dorsolateral) right supplementary motor area	15 12 57	15	5.72954
	Cluster 3	right median cingulate	12 15 33	11	5.42893
	Cluster 4	lest inferior frontal gyrus (orbital part) left middle frontal gyrus (orbital part)	-33 42 -3	9	6.22811
Left Amygdala	Cluster 1	right middle frontal gyrus (orbital part) rights superior frontal gyrus (orbital part)	30 51 -3	9	4.76529

All were corrected for multiple comparisons with the Gaussian Random Field (GRF) theory ($p < 0.001$ at voxel-level, $p < 1$ at cluster level, two-tailed). Only the FC between right amygdala and Cluster 1 was statistically significant ($p < 0.01$ at cluster level).

Table S5. FC intensity and significant level before and after intervention in all participants and the acute exercise group.

	Pre			Post		
	t	z	p	t	z	p
ALL	8.99	5.77	8.014E-09	6.71	4.90	9.592E-07
AG	5.72	3.82	1.33E-04	7.96	4.50	6.8913E-06

The functional connection between the amygdala and the right orbitofrontal cortex was examined by a one sample t test.

Table S6. Correlations between changed FC, affect scores, and HR.

		FC	Tension	Anger	Fatigue	Depression	Vigor	Confusion	Esteem	TMD	PA	NA
**Baseline_HR**	r	-0.180	0.449	0.379	-0.023	0.010	-0.004	-0.004	0.438	0.063	0.504	0.254
	p	0.575	0.143	0.224	0.944	0.976	0.990	0.989	0.155	0.845	0.095	0.426
Exercise_HR	r	0.018	-0.131	-0.097	-0.546	-0.103	0.107	-0.135	0.209	-0.333	0.382	-0.097
	p	0.956	0.684	0.765	0.066	0.749	0.742	0.676	0.514	0.291	0.220	0.763

The correlation between HR (Baseline HR and Exercise HR) and FC alteration as well as the correlation between HR and affect scores changes.

FC, the change of functional connectivity between amygdala and right orbitofrontal cortex.
